# Synthesis, characterization, and photophysical and fluorescence sensor behaviors of a new water-soluble double-bridged naphthalene diimide appended cyclotriphosphazene

**DOI:** 10.55730/1300-0527.3613

**Published:** 2023-07-27

**Authors:** Süreyya Oğuz TÜMAY, Serkan YEŞİLOT

**Affiliations:** Department of Chemistry, Faculty of Science, Gebze Technical University, Kocaeli, Turkiye

**Keywords:** Cyclotriphosphazene, double-bridged, fluorescence, sensor, photophysical properties

## Abstract

A new water-soluble template of double-bridged naphthalene diimide appended cyclotriphosphazene was prepared, and its photophysical and sensor behaviors were evaluated. The characterization of novel double-bridged naphthalene diimide appended cyclotriphosphazene (**6**) was carried out by NMR (^1^H, ^13^C, ^31^P) and mass spectroscopies. The photophysical behaviors of compound 6 were evaluated by UV-Vis absorption and fluorescence spectroscopies in various solvent systems and different concentrations. As an application for usability of the obtained water-soluble template in different applications, the fluorescence sensor property of compound 6 was investigated in the presence of many different competing species (organic acids, saccharides, nitroaromatic compounds, anions, and metal cations). The results obtained showed that compound 6 had selectivity against only the nitroaromatic species among the competing species tested.

## 1. Introduction

As a significant member of the heterocyclic inorganic ring, cyclophosphazenes contain (N = PX2)n repeating part and they were extensively applied in various applications such as biomedical research [1], liquid crystals [2], catalysis [3], and flame retardancy [4]. Especially in the last decade, cyclotriphosphazene core takes attention to sensor applications due to their optical inertness and chemical stability when compared with organic corresponds [5]. The inorganic-organic materials that contain cyclotriphosphazene core are important candidates for luminescence-based applications not only for stability or optical inertness but also because the appended functional moieties are reflected in 3 dimensions, thereby producing a rigid spherical core that can be used for coordination or interaction with competitive species in sensor applications. In addition, resulting rigid cores have been used for the application of electroluminescent devices because it is known that this type of materials can demonstrate amorphous properties [6,7]. The water-solubility of fluorescence compounds is a desired property, especially in sensor applications because the analysis medium in environmental or biological samples consists of water [8]. Although cyclotriphosphazene cores demonstrate extraordinary chemical and physical properties, they mainly do not dissolve in water, whereas they are highly soluble in organic solvents. When considering the effect of water on fluorescence intensity, the weak water solubility of cyclotriphosphazene-based fluorescence materials limited their luminescence applications [8]. For cyclotriphosphazene, having reactive six Cl-atoms, researchers have tried to overcome this problem recently by modifying phosphazenes with water-soluble groups together with fluorescent dyes [9–12]. In this manner, cyclotriphosphazene-based fluorescent water-soluble materials have been synthesized in the last two decades to be used in different applications [13–16]. Cyclicphosphazenes can be modified by nucleophilic reaction with mono-, bi-, and polydentate reagents to obtain mono- to hexasubstituted or rarely bridged products [17–20].

Naphthalene diimides (NDI) are one of the extensively used p-conjugated molecules when considering their extraordinary electrical and optical behaviors [21, 22]. The reason naphthalene diimides are so interesting can be attributed to their oxidative stability, appropriate self-assembly, excellent thermal stability, solubility in organic solvents, reversible redox properties, high photoluminescence quantum yields, good charge mobility, and high electron affinity [23]. Owing to these superior features, a wide range of applications like nanochemistry [24], electronics [25], or sensors [26] use NDIs as a precursor. In addition, the high melting points, redox activity, planar, neutral, and chemically stable structures of NDIs make them useful in electronic materials, supramolecular interactions, and chromophores. 1,4,5,8-Naphthalene tetracarboxylic dianhydride (NTCDA) is the main precursor for the synthesis of a variety of NDI molecules. Nowadays, owing to the imide nitrogen and modification of the naphthyl core, numerous NDI derivatives can be synthesized using NTCDA as a main precursor [27].

**Figure 1:**
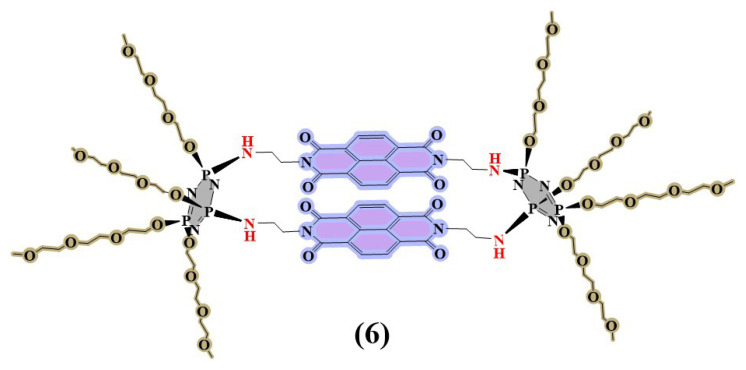
Chemical structure of compound 6.

In this report, we presented the molecular design, synthesis and characterization of new double-bridged naphthalene diimide appended cyclotriphosphazene (**6**) (Figure 1) which was prepared via nucleophilic substitution reactions of NDI-based precursor (**3**) and water-soluble cyclotriphosphazene core (**5**) in the presence of NaH. After full characterization of the new water-soluble compound **6**, its photophysical and sensor behaviors were evaluated by UV-Vis and fluorescence spectroscopies.

## 2. Materials and methods

Ethylenediamine (≥99%), di-*tert*-butyl dicarbamate (99%), 1,4,5,8-naphthalenetetracarboxylic dianhydride, phosphonitrilic chloride trimer (cyclotriphosphazene, 99%), triethylene glycol monomethyl ether (TEGME, 95%), sodium hydride dispersion (NaH, 60% in mineral oil), 2,5-dihydroxybenzoic acid (98%), analytical grade of all solvents, silica gel and silica gel plates which were coated with F_254_ indicator for column chromatography and TLC were purchased from Merck (Germany). Any extra purification procedure was applied for commercial chemicals except for cyclotriphosphazene, NaH, and THF. The distillation procedure was applied for the purification of THF by alloy (sodium/potassium), whereas washing and crystallization procedures with n-hexane were carried out for the purification of NaH and cyclotriphosphazene, respectively. The stock solutions of competitive species were prepared using nitrate salt of metal ions, sodium salts of anions and analytical grades of organic acids, NACs, and saccharides. The Bruker Daltonics Microflex MALDI-TOF spectrometer (USA) and 500 MHz NMR (Varian INOVA, USA) were used for obtaining mass and NMR (^13^C, ^1^H and ^31^P) spectra of the compound **6**, respectively. The MALDI-TOF spectrum was recorded with dithranol as a matrix whereas D_2_O and methanol-d_4_ were used as a solvent for NMR spectra. The UV-Vis absorption and fluorescence measurements of compound **6** were recorded with Shimadzu 2101-UV spectrophotometer (Japan) and Varian Cary Eclipse spectrofluorometer (USA), respectively. The spectroscopic cuvette which does not absorb in the UV-Vis region (quartz, 1 cm path length) was used for the evaluation of sensor and photophysical behaviors.

### 2.1. Synthesis of compound 6

**Figure 2:**
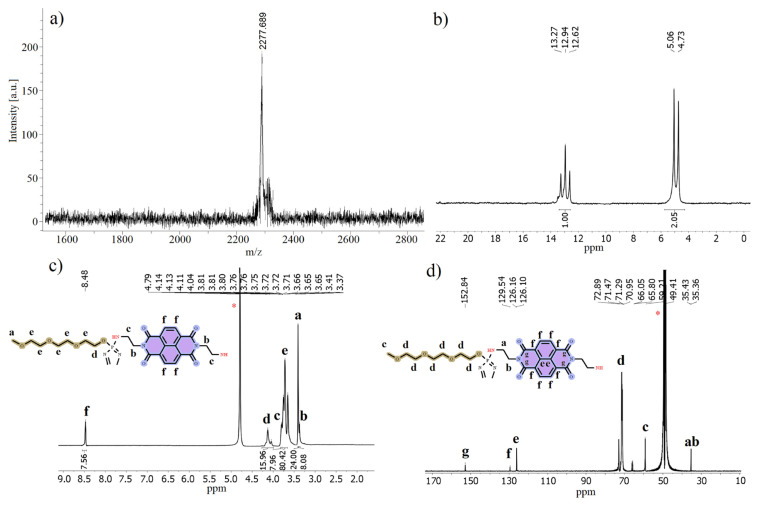
a) MALDI-TOF, b) ^31^P NMR, c) ^1^H NMR, and d) ^13^C NMR of compound 6 (dithranol used as a matrix for MALDI-TOF, methanol-d_4_ used for ^31^P NMR and D_2_O used for ^1^H/^13^C NMR).

*Tert*-butyl (2-aminoethyl)carbamate (**1**), compounds **2**–**5** were synthesized and characterized according to previous reports [17, 20, 28, 29]. The characterization data (MALDI-TOF, ^31^P, ^1^H, and ^13^C NMR) of compound **6**, which were in good agreement with the predicted chemical structure of compound **6**, are given in Figure 2.

#### 2.1.1. cis-2,4-(2,7-bis(2-(methylamino)ethyl)naphtalenediimide)-bridge-octakis(methyltri-glycol)-cyclotriphosphazene (6)

Compound **5** (180 mg, 0.209 mmol) was dissolved in 10 mL of acetonitrile at 0 °C under inert atmosphere and then NaH (51 mg, 1.257 mmol, 60%) was added to the resulting solution and stirred continuously at 0 °C. Next, compound **3** (261 mg, 0.732 mmol) suspended in 10 mL of acetonitrile was added onto the resulting mixture and the reaction mixture was regularly followed by thin layer chromatography. After the compound **5** was finished, the reaction mixture was filtered through a G4 filter and the solvent of the filtrate was partially removed by applying vacuum. The remaining reaction mixture was purified by the preparative thin layer column chromatography technique using methanol as the mobile phase. The obtained product (**6**) had a yellow oily appearance (10 mg, yield: 2.1%). ^31^P NMR (202 MHz, methanol-d_4_): δ = 12.94 ppm [t, > P(C_7_H_15_O_4_)_4_] δ = 4.90 ppm [d, > P((C_7_H_15_O_4_)NH)_4_]. ^13^C NMR (500 MHz, D_2_O), δ (ppm): 152.84, 129.54, 126.16, 126.10, 72.89, 71.47, 71.29, 70.95, 66.05, 65.80, 59.21, 49.41, 35.43, 35.36. ^1^H NMR (500 MHz, D_2_O), δ (ppm): 8.48 (s, 8H), 4.10 (m, 16H), 3.81 (t, J = 3.9 Hz, 8H), 3.76–3.65 (m, 80 H), 3.41 (s, 24H), 3.37 (t, J = 3.7 Hz, 8H). MALDI-TOF; [M + H]^+^: 2277.689 m/z, (calc. C_92_H_148_N_14_O_40_P_6_, 2276.10 g/mol). Elemental analysis; found: C 48.26, H 6.45, N 8.55%; C_92_H_147_N_14_O_40_P_6_ requires C 48.57, H 6.51, N 8.62%.

### 2.2. Photophysical calculations

The Stokes shift (Δλ) and fluorescence quantum yield (∅_F_) for compound **6** were evaluated as photophysical parameters. The quinine sulfate (∅_F_ = 0.54) which dissolved in 0.1 M H_2_SO_4_ solution was applied as a standard for fluorescence quantum yield calculation using the comparative method (Eq. 1) [30, 31].


(Eq. 1) $$\emptyset F=\emptyset F_{Std}\frac{F.A_{Std}.n^{2}}{F_{Std}.A.n_{Std}^{2}}$$

In this calculation, ∅_F_, F, A, ∅_Fstd_, F_std_, and A_std_ demonstrate fluorescence quantum yields, fluorescence band areas, and absorbance values (at excitation wavelength) of sample and quinine sulfate, respectively. Also, the refractive indices of the sample and quinine sulfate are given as n and n_std_, respectively.

## 3. Results and discussion

The preparation route of the target water-soluble cyclotriphosphazene-based compound **6** is given in Scheme. The water-soluble double-bridged cyclotriphosphazene platforms have been recently introduced to the literature [17, 20]. In these reports, obtained water-soluble double-bridged cyclotriphosphazenes included electron-rich fluorophores, which were used as fluorescent sensors for the detection of TNT and NACs [17, 20]. According to the information we obtained from these reports, we introduced a new double-bridged naphthalene diimide appended cyclotriphosphazene. Naphthalene diimide bridges, which are part of this structure, are used as an optical signal unit and also, unlike the electron-rich bridges [17, 20], the effect of electron-deficient naphthalene diimide structure on sensor properties was investigated. In presenting structure, TEGME fragments used as a solubilizing agent were used for their water-soluble properties [32, 33]. The mono *tert*-boc protected ethylenediamine (**1**) and its NDI derivative (**2**) were synthesized and characterized according to the literature [28]. In addition, deprotection of compound **2** was applied by following the literature in order to obtain compound **3** [29]. Briefly, the ethylenediamine was reacted with di-*tert*-butyl dicarbamate at 0 °C in chloroform. The compound **1** was obtained via extraction and used for the next steps without further purification. The NDI derivative (**2**) was prepared with the reaction of NDI with compound **1** in the presence of DMF at 75 °C. The column chromatography was applied in order to purify compound **2**. The deprotection of compound **2** was carried out in the presence of TFA and compound **3** was obtained without further purification. The water-soluble phosphazene platform named cis-tetrakis(methyltriglycol)-cyclotriphosphazene (**5**) was synthesized according to the literature [20]. Finally, the target novel double-bridged naphthalene diimide appended cyclotriphosphazene (**6**) was prepared in ACN via the nucleophilic substitution reaction compounds **3** and **5** where NaH was used as a base.

The chemical structure of compound **6** was elucidated by examining the information obtained from the mass, ^31^P, ^1^H, and ^13^C NMR spectra. Mass analysis of compound **6** was performed using a dithranol matrix by MALDI-MS technique (Figure 2). In the obtained mass spectrum (Figure 2a), the [M + H]^+^ peak was observed at 2277.689 m/z and was in agreement with the theoretically calculated value (C_92_H_148_N_14_O_40_P_6_, 2276.10 g/mol). The proton-decoupled ^31^P NMR (500 MHz) spectrum of compound **6** was recorded into methanol-d_4_ at room temperature which demonstrated that δ = 4.90 ppm [ > P((C_7_H_15_O_4_)NH)_4_] doublet peak and δ = 12.94 ppm [ > P(C_7_H_15_O_4_)_4_] triplet peak were obtained. It was figured out that the structure has an AX_2_ spin system (Figure 2b). The phosphorus atoms with A_2_X spin system of compound **5** [17], observed as a doublet peak at δ = 24.234 ppm and a triplet peak at δ = 12.169 ppm, turn into AX_2_ spin system for compound **6** as a result of the reaction and change in their chemical shifts. This data shows that while the chemical environment of the two phosphorus atoms in the structure of compound **6** is the same, the third phosphorus atom has a different chemical environment from the others and that the phosphorus atoms in compound **5** and compound **6** have different chemical environments from each other. The proton-decoupled ^31^P NMR spectrum supports the proposed structure of compound **6**. The ^1^H NMR (500 MHz) spectrum of compound **6** was recorded in D_2_O at room temperature and the methyl protons in the compound were observed as a single peak at δ = 3.408 ppm, while the methylene protons in the -HN-CH_2_-CH_2_-NH-group were obtained at δ = 3.80 ppm and δ = 3.37 ppm as triplet peaks. Other methylene protons were observed at δ = 4.14–3.65 ppm, and aromatic protons were observed at δ = 8.48 ppm (Figure 2c). The ^13^C NMR (500 MHz) spectrum of compound **6** was recorded in D_2_O at room temperature, and -HN-CH_2_-CH_2_-NH- carbons were observed at δ = 35.43 ppm and δ = 35.36 ppm, methyl carbons and other methylene carbons were observed as single peaks at δ = 59.21 ppm and at δ = 72.89–65.80 ppm, respectively. The aromatic carbon atoms in the structure were obtained at δ = 129.54–126.10 ppm while carbonyl group carbon atoms were observed at δ = 152.84 ppm (Figure 2d).

**Figure 3:**
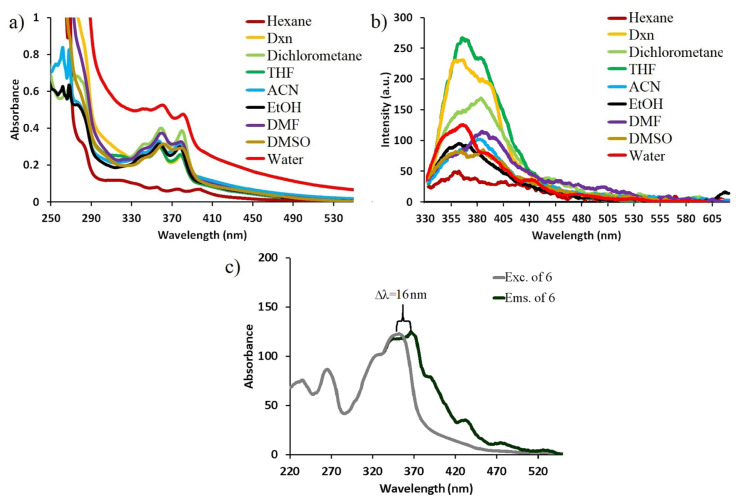
a) UV-Vis absorption, b) fluorescence spectra of 3.2 × 10^−^^4^ M compound 6 in different solvents and **c)** Stokes shift in water (λ_exc_ = 320 nm).

The photophysical properties of the target novel double-bridged naphthalene diimide appended cyclotriphosphazene (**6**) were evaluated by UV-Vis and fluorescence spectroscopy using spectroscopic quartz cuvettes at 25 °C. UV-Vis spectra of 3.2 × 10^−4^ M compound **6** in different solvents such as hexane, 1,4-dioxane, THF, dichloromethane, acetonitrile (ACN), ethanol (EtOH), dimethylformamide (DMF), dimethyl sulfoxide (DMSO), and water were examined. As seen in Figure 3a, the absorption maxima of **6** were obtained at ≈260 and 360 nm. The absorption properties observed in compound **6** were similar to those of the fluorophore group (naphthalene diimide) in its structure, and these absorption properties can be attributed to π–π* transitions of naphthalene diimide fluorophores [34]. This is due to the fact that phosphazene compounds are optically inert in the UV-Vis region and optical properties of this core can be adjusted according the fluorophores added to their structures [12].

In the fluorescence spectrum of 3.2 × 10^−4^ M compound **6** was examined with the same solvents, the fluorescence emission maximum of compound **6** was determined at 356–385 nm (Figure 3b). As observed in Figure 3b, the fluorescence signals of compound **6** obtained in different solvents are the same as the fluorescence signals of monomeric NDI moieties [34], and the fluorescence emissions of the compound **6** were stable against varying solvent systems. The Stokes shift of compound **6** in water was determined as 16 nm, whereas fluorescence quantum yield was determined as 0.08 (Figure 3c).

**Figure 4:**
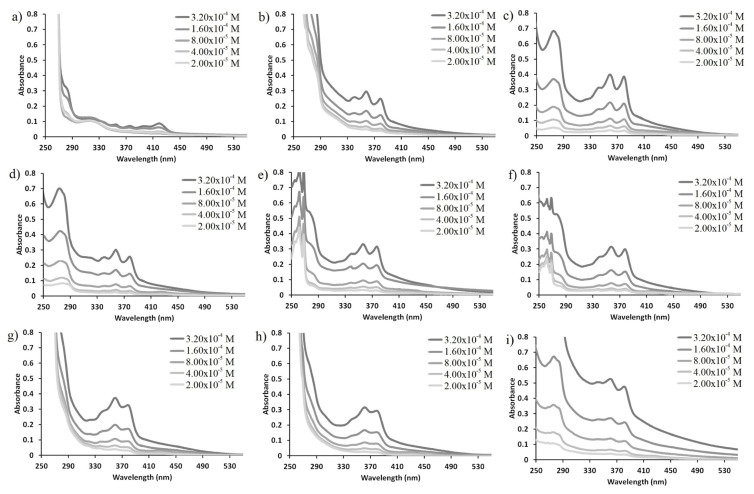
UV-Vis spectra of compound 6 at varying concentrations in different solvents a) hexane, b) dioxane, c) dichloromethane, d) THF, e) ACN, f) EtOH, g) DMF, h) DMSO and i) water.

**Figure 5:**
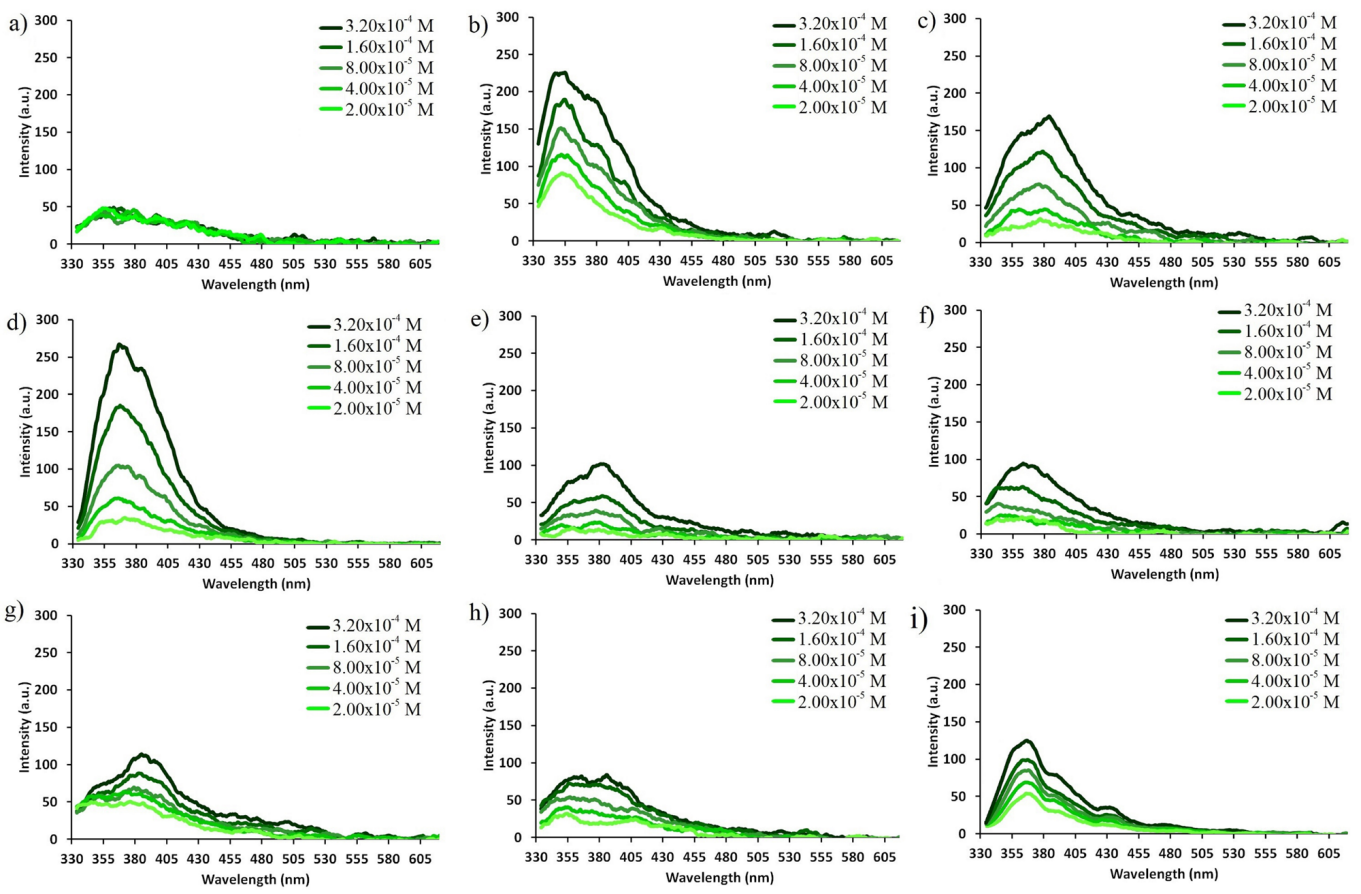
Fluorescence spectra of compound 6 at varying concentrations in different solvents a) hexane, b) dioxane, c) dichloromethane, d) THF, e) ACN, f) EtOH, g) DMF, h) DMSO and i) water (λ_exc_ = 320 nm).

As it is well-known, the optical properties of fluorophore groups can change depending on the applied concentration [35]. Therefore, UV-Vis and fluorescence spectra of compound **6** at varying concentrations in different solvents were investigated (Figures 4 and 5). It showed that the optical signals of compound **6** change in direct proportion with decreasing concentration without any appreciable shift in wavelengths. This indicates that there is no intramolecular or intermolecular interaction between the NDI groups in compound **6** not only in ground state but also in excited states [12, 17].

**Figure 6:**
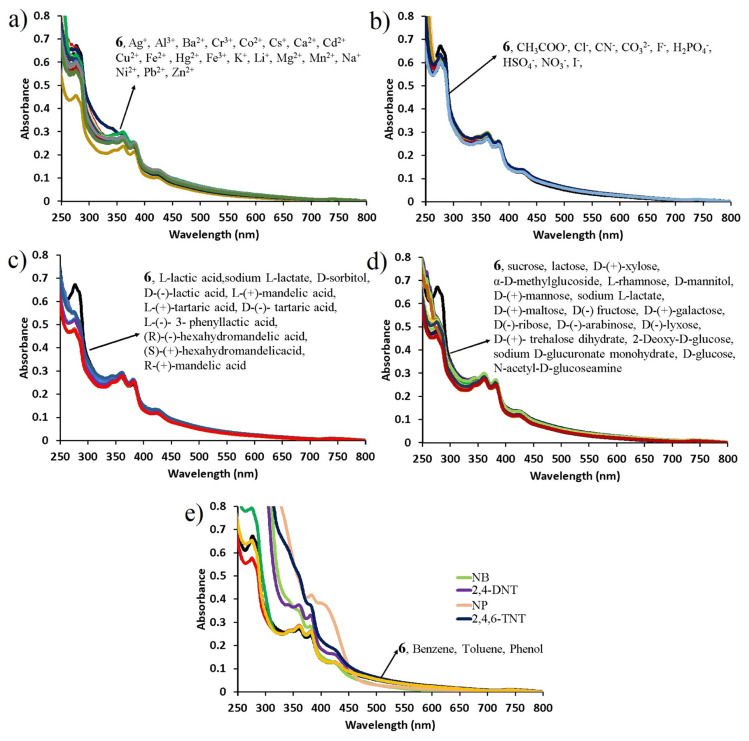
UV-Vis absorption spectra of 1.6 × 10^−4^ M compound 6 after addition of a) 100 equivalents of metal cations, b) 100 equivalents of anions, c) 500 equivalents of organic acid, d) 500 equivalents of saccharides, and e) 15 ppm aromatic/nitroaromatic compounds to the aqueous solution of compound 6.

The fluorescence sensor properties of compound **6** against metals, anions, saccharides, organic acids, and aromatic/nitroaromatic compounds were evaluated by the UV-Vis absorption and fluorescence spectroscopies. The UV-Vis absorption signal changes of 1.6 × 10^−4^ M compound **6** against 100 equivalents of metal ions (Ag^+^, Cs^+^, K^+^, Li^+^, Na^+^, Ba^2+^, Ca^2+^, Cd^2+^, Co^2+^, Cu^2+^, Fe^2+^, Hg^2+^, Mg^2+^, Mn^2+^, Ni^2+^, Pb^2+^, Zn^2+^, Al^3+^, Cr^3+^, Fe^3+^), 100 equivalents of anions (Cl^−^, F^−^, I^−^, CO_3_^2−^, NO_3_^−^, SO_4_^2−^, HSO_4_^−^, CN^−^, H_2_PO_4_^−^), 500 equivalents of saccharides and organic acids (sucrose, lactose, D-(+)-xylose, α-D-methylglycoside, L-rhamnose, D-mannitol, D-(+)-mannose, sodium L-lactate, D-(+)-maltose, D(−) fructose, D-(+ )-galactose, D(−)-ribose, D-(−)-arabinose, D(−)-lyxose, D-(+)-trehalose dihydrate, 2-deoxy-D-glucose, D-glucose, D-sorbitol, sodium D-gluconate monohydrate, N-acetyl-D-glucosamine, L-lactic acid, D-(−)-lactic acid, L-(+)-mandelic acid, R-(+)-mandelic acid, D-(−)-tartaric acid, L-(+)-tartaric acid, L-(−)-3-phenyllactic acid, (R)-(−)-hexahydromandelic acid, (S)-(+)-hexahydromandelic acid) and 15 ppm aromatic/nitroaromatic compounds (benzene, toluene, phenol, nitrophenol, nitrobenzene, 2,4-DNT, TNT) are depicted in Figure 6.

As seen in Figure 6, metal ions, anions, saccharides, and organic acids added in the aqueous solution of compound **6** had no effect on its maximum absorption at 360 nm, while the addition of nitroaromatic compounds increased the maximum absorption wavelength of compound **6** by ≈1.5 (Figure 6). As a result of the selectivity study performed with UV-Vis spectroscopy, it was observed that compound **6** showed a moderate selectivity against only nitroaromatic compounds among tested competitive species and that the absorption properties caused by π - π* transitions of the fluorophore were changed only by adding nitroaromatic compounds. However, no selectivity was observed for compound **6** among aromatic compounds containing nitro groups (nitrophenol, nitrobenzene, 2,4-DNT, and TNT). These changes in UV-Vis absorption spectra of compound **6** with the addition of NACs can be attributed to the electronic reorganization and H - bonding between compound **6** and NACs, which is in agreement with the literature [36, 37].

**Figure 7:**
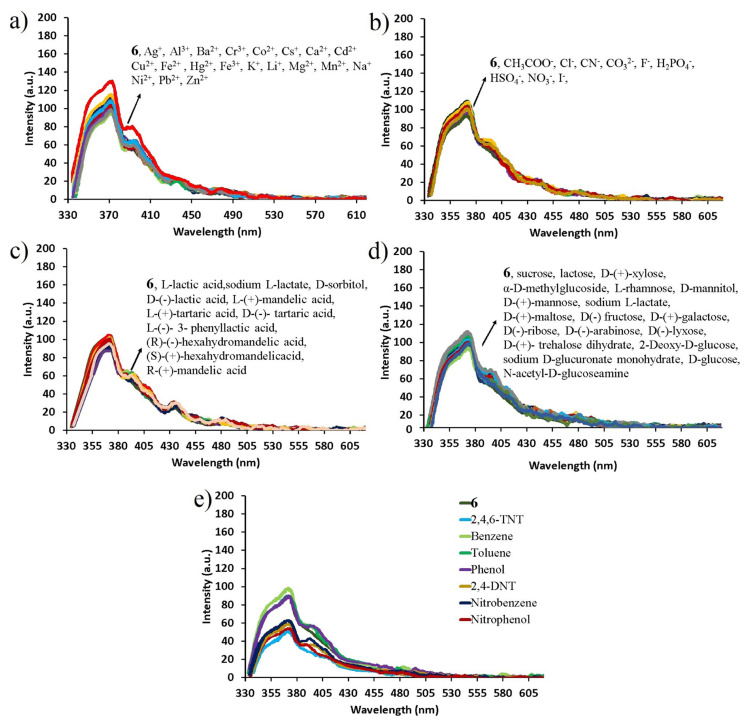
Fluorescence spectra of 1.6 × 10^−4^ M compound 6 after addition of a) 100 equivalents of metal cations, b) 100 equivalents of anions, c) 500 equivalents of organic acid, d) 500 equivalents of saccharides, and e) 15 ppm aromatic/nitroaromatic compounds to the aqueous solution of compound 6. (λ_exc_ = 320 nm).

After examining the UV-Vis absorption responses of compound 6 against the competing species, the selectivity of the compound 6 against metals, anions, saccharides, organic acids, and aromatic/nitroaromatic compounds was examined by fluorescence spectroscopy under the same analytical conditions with absorption studies (Figure 7). In all fluorescence emission measurements, compound **6** was excited at wavelengths of 320 nm. A similar situation was observed in the fluorescence emission response of 1.6 × 10^−4^ M compound **6**. As seen in Figure 7, metal cations, anions, saccharides, and organic acids added in the aqueous solution of compound **6** had no effect on the maximum fluorescence emission at 368 nm, while the addition of nitroaromatic compounds caused max. 47.45% quenching at 368 nm.

As a result of the selectivity study performed by fluorescence spectroscopy, it was observed that compound **6** showed a moderate selectivity only against NACs among the competing species and its fluorescence properties were changed only by adding NACs, but no selectivity was observed among NACs. In the reports introduced by our research group, the fluorescence quenching rate was 85.52% and 57.20% for anthracene- and naphthalene bridged water-soluble cyclotriphosphazenes [17, 20]. The reason why the change in fluorescence emission spectra is less (47.45%) under the same condition after the addition of NACs in compound **6** is most probably that the electron-deficient nature of NDI groups in compound **6** prevents effective interaction with NACs and reduced selectivity and sensitivity [27, 38, 39]. In addition, the efficient electron transfer occurring from electron-rich fluorophores (anthracene and naphthalene) to electron-deficient NACs and H-bond between them led to fluorescence quenching more efficiently [17, 20]. With this study, we not only provide evidence that sensors containing electron-rich groups can detect NACs, but we also show that the optical properties of new water-soluble double-bridged cyclotriphosphazene template can be modified according to analytes with desired properties.

## 4. Conclusion

In conclusion, we introduced the new water-soluble double-bridged template of cyclotriphosphazene platform modified with naphthalene diimide (**6**). The synthesis of compound **6** can be performed by simple nucleophilic substitution reaction, and chemical characterization was performed by spectroscopic techniques (mass, ^31^P, ^13^C, and ^1^H NMR). According to photophysical investigations, the absorption properties of compound **6** were similar to those of naphthalene diimide in its structure, and absorption properties can be attributed to π – π* transitions of naphthalene diimide fluorophores which is expected due to the optical inertness of phosphazenes in the UV-Vis region. In addition, fluorescence properties of compound **6** in different solvents can be attributed to monomeric emission properties of monomeric NDI moieties. The fluorescence sensor behaviors of compound **6** revealed that “turn-off” signal change can be obtained against NACs with moderate selectivity between other tested competitive species (metal cations, anions, saccharides, aromatic compounds, and organic acids) in fully aqueous media. According to all measurements, the presented double-bridged naphthalene diimide appended cyclotriphosphazene (**6**) can be potentially a candidate for important applications such as ion-channels by ligand gating, host–guest complexes, and sensing aromatic systems.

## Figures and Tables

**Scheme:**
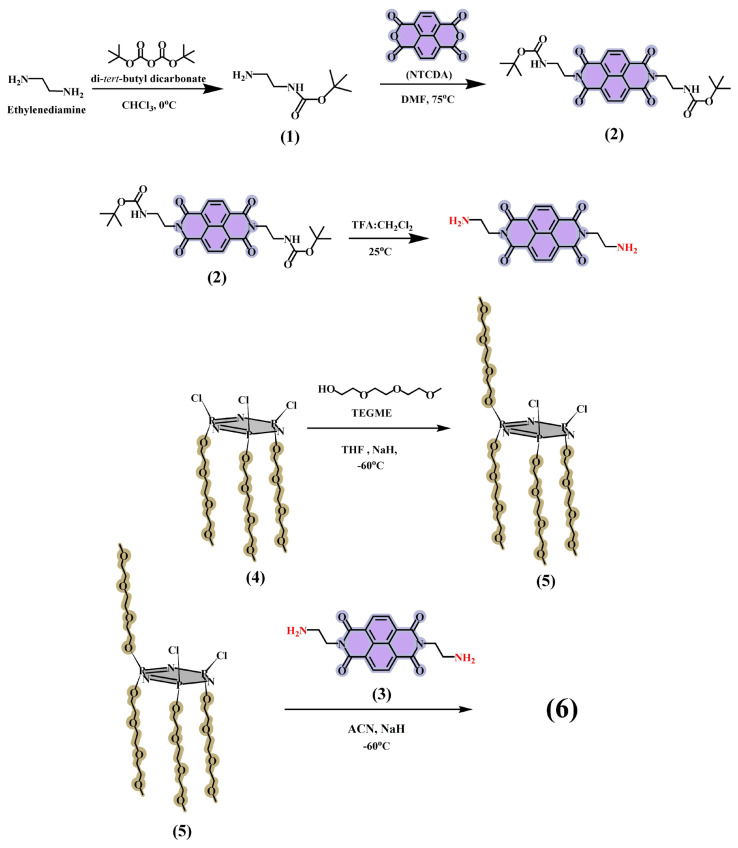
The preparation route of the target water-soluble cyclotriphosphazene-based compound 6.
